# Significant enhanced uranyl ions extraction efficiency with phosphoramidate-functionalized ionic liquids via synergistic effect of coordination and hydrogen bond

**DOI:** 10.1038/s41598-017-15899-0

**Published:** 2017-11-16

**Authors:** Xiang Xie, Zhen Qin, Yao He, Penghui Xiong, Zeng Huang, Yiwu Mao, Hongyuan Wei, Liangang Zhuo

**Affiliations:** 1 Institute of Nuclear Physics and Chemistry, China Academy of Engineering Physics, Mianyang, 621900 China; 2 Institute of Materials, China Academy of Engineering Physics, Mianyang, 621900 China

## Abstract

The influence of the linking group between the phosphoryl and bridging moieties in phosphoryl-containing task-specific ionic liquids (TSILs) on the extraction of uranyl ions was experimentally and theoretically investigated. A novel phosphoramidate-based TSIL with an amine group as the linking moiety resulted in a higher uranyl ion extraction efficiency compared with that of other phosphoryl-based TSILs. A distribution ratio of 4999 ± 51 can be achieved for uranyl ions. The uranyl ions (76.7 ± 1.5%) were stripped from the loaded ionic liquid phase in a single stage using 0.05 M diethylenetriamine pentaacetic acid in a 1.0 M guanidine carbonate solution. The extraction stoichiometry of the uranyl ions was determined by a slope analysis of the extraction data. Furthermore, the fundamental nature of the interaction between the phosphoramidate-based TSIL and uranyl ions was theoretically studied for the first time. The theoretical calculations demonstrated that the synergistic effect of the complexation interaction and H-bond formation between the phosphoramidate-functional ionic liquid and uranyl nitrate led to the higher extraction efficiency. These results provide a basis for rational design, synthesis and potential applications of novel TSILs for uranyl extraction.

## Introduction

Nuclear energy is going to one of the major sources of energy due to increasing power demands. Uranium is an important element in most of the currently operating nuclear reactors. The recovery of uranium from spent nuclear fuel is essential for the sustainable development of nuclear energy^1,2^. Solvent extraction is the most commonly used process to separate uranium from acidic media with neutral ligands in the nuclear industry^3^. However, traditional liquid-liquid extraction methods using organic solvents suffer from disadvantages, including high volatility, high flammability and toxicity^4^.

Room temperature ionic liquids (RTILs) are considered environmental friendly and “green” alternative diluents relative to molecular solvents because of their negligible vapor pressure, high thermal stability, high electrical conductivity and low flammability^5–8^. In recent years, RTILs have been employed as diluents for the recovery of uranium from acidic feed conditions^9–15^.

To increase the solubility of metal complexes in ionic liquid phases, task-specific ionic liquids (TSILs), or functionalized ionic liquids, which are usually composed of a functional group covalently tethered to the cationic or anionic segment of RTILs, have been developed^16,17^. Recently, a series of TSILs containing 2-hydroxybenzylamine^18^, phosphate^20–25^, carboxyl^25^, diglycolamide (DGA)^27–31^, CMPO^31,32^, malonamide^33^, hydrogen phthalate^34^, amidoxime^35^ or betainium groups^37–39^ have been reported for the dissolution and extraction of lanthanides and actinides. Of the various TSILs that have been tested for lanthanide/actinide extraction, TSILs bearing phosphoryl or alkyl phosphate groups, which are analogous to TBP, have been considered as alternatives to conventional ionic liquid systems containing TBP for the extraction of uranyl ions from nitric acid media. However, in most cases, TBP-based TSILs have not produced better results than solvent systems containing TBP in an ionic liquid, and sometimes TSILs have shown poor extraction efficiencies compared to those of traditional molecules. A similar, relatively inefficient extraction was observed with CMPO-based TSILs. Ouadi *et al*.^19^ synthesized a novel class of TSILs based on quaternary ammonium cations with phosphoryl groups to extract uranyl ions. Their preliminary results suggested that phosphoramide-functionalized ionic liquids have high distribution ratios for uranyl ions. However, the main reason for the large extraction coefficient of the phosphoramide-containing TSIL compared to those of the other two TSILs is not well understood. Based on the chemical structure of the reported TSILs, there are two possible reasons for the high extraction coefficient. One reason may be the amine (NH) linking group between the phosphoryl moiety and the bridging moiety, which can provide an additional active site in the phosphoramide-functionalized ionic liquid for uranyl extraction. The other possible reason is that the propyl bridging moiety between the phosphoramide and quaternary ammonium cation can adopt a more favorable conformation than those in the other TSILs.

**Figure 1:**
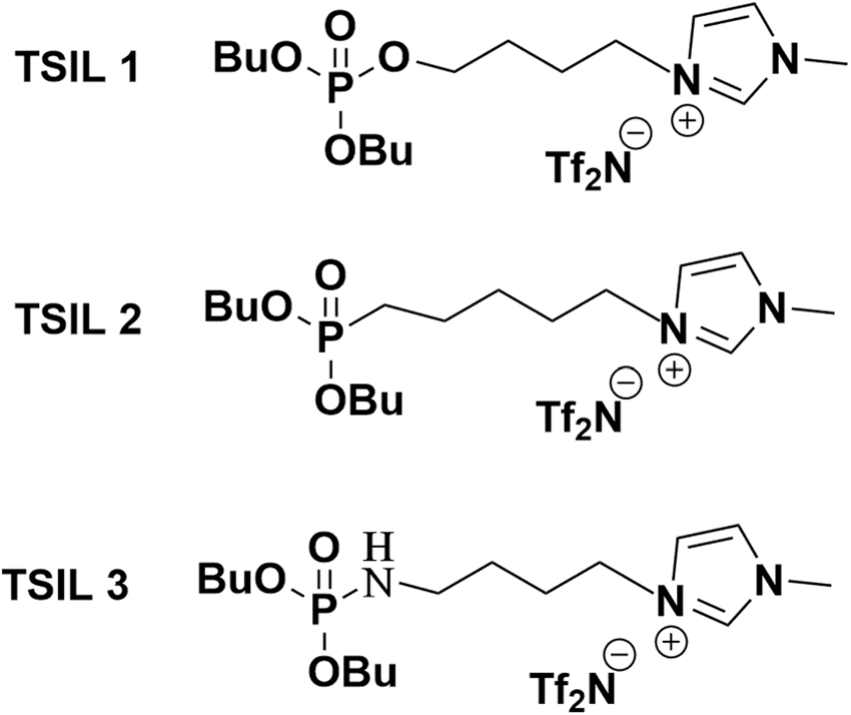
Structures of the phosphoryl-functionalized TSILs.

Therefore, investigating the important role of the linking group between the phosphoryl moiety and bridging moiety in phosphoryl-containing TSILs in the extraction of uranyl ions is important. For this purpose, we synthesized a class of TSILs with phosphoryl groups and incorporated different linking groups, including ether (TSIL **1**), alkyl (TSIL **2**) and amine (TSIL **3**) groups, with the butyl bridging moieties of the imidazolium cations, as shown in Fig. 1. The role of the linking group in the TSILs on the extraction efficiency for uranyl ions was comprehensively investigated by studying the distribution ratios (*D*) and extraction kinetics. Additionally, density functional theory (DFT) calculations were used to investigate the differences in the coordination modes.

## Results and Discussion

### Synthesis of phosphoryl-functionalized imidazolium-based ionic liquids

**Figure 2:**
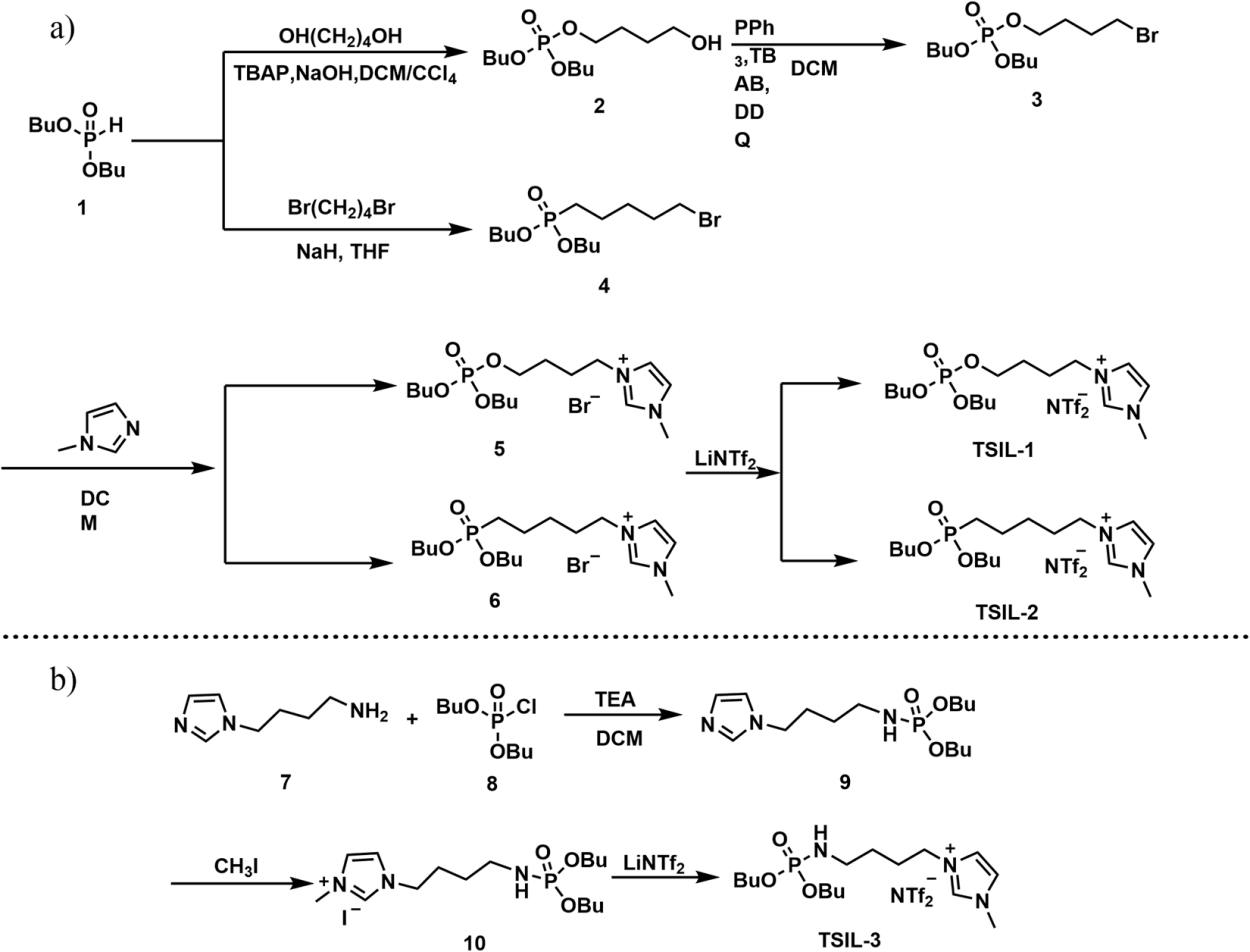
(**a**) Synthesis routes for TSIL 1 and TSIL 2. (**b**) Synthesis route for TSIL 3.

The synthesis of TSIL **1** and TSIL **2** is summarized in Fig. 2a. TSIL **1** was synthesized as described in the literature with some modifications^22^. Phosphine oxide moieties were grafted onto imidazolium cations via a four-step synthesis starting with dibutyl phosphate and alkyl alcohol to afford compound **2**, which was then bromized with the assistance of PPh_3_ to yield compound **3**. Compound **3** was subsequently reacted with 1-methylimidazole in DCM for 48 h to afford the imidazolium bromide compound **5**, which was finally transformed into TSIL **1** with LiTf_2_N via a simple anion exchange reaction.

We developed a simple synthetic approach with a high yield for the preparation of phosphonium-based TSIL **2**. Commercially available dibutyl phosphate was treated with 1,4-dibromobutane in THF using NaH as the deprotonation agent to afford compound **4**. Compound **4** was subsequently reacted with 1-methylimidazole in DCM for 48 h to afford the imidazolium bromide compound **6**, which was finally transformed into the TSIL **2** with LiTf_2_N via a simple anion exchange reaction. This new synthetic route simplified the method and resulted in a high yield of carbon chain phosphonium, and a further methodology study is ongoing in our group.

TSIL **3** was synthesized via a three-step reaction that started with 1-(4-aminobutyl) imidazole and dibutyl chlorophosphate to obtain the corresponding phosphoramide and was followed by quaternization of the imidazole with methyl iodide^39^, as shown in Fig. 2b. A metathesis with LiTf_2_N converted the iodide into bis(trifluoro-methanesulfonyl)amide. All the TSILs synthesized in this work were liquids at 25 °C.

### Physical properties of TSILs

**Figure 3:**
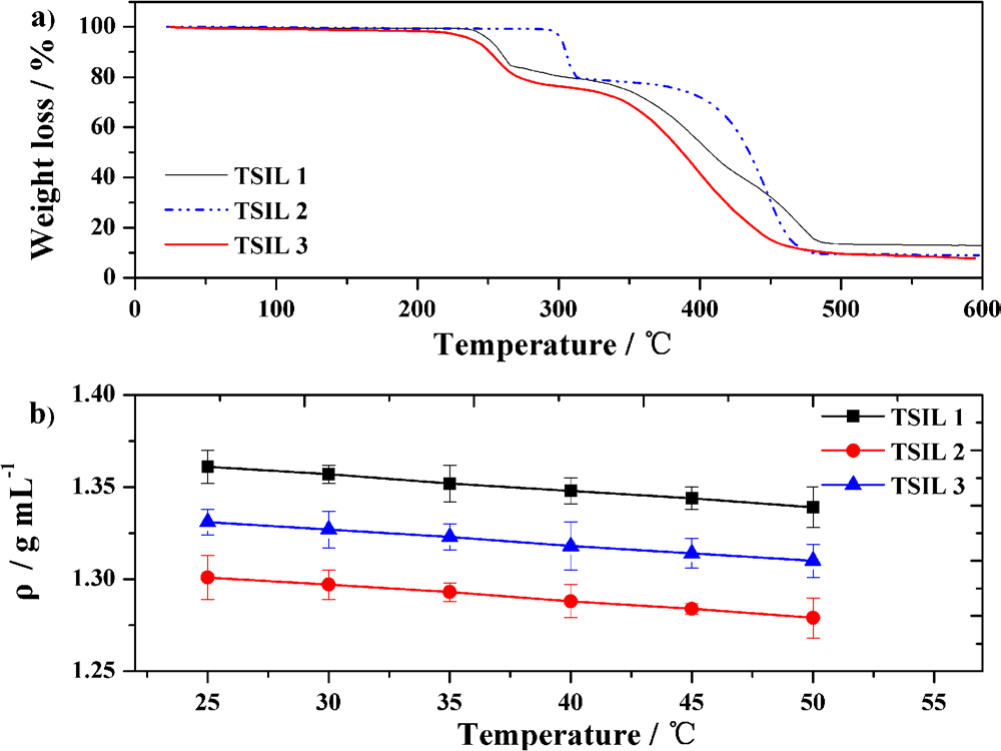
(**a**) TGA of TSILs 1-3. (**b**) Temperature dependence of the density for TSILs 1-3.

To investigate the thermal stability of the obtained TSILs, a thermogravimetric analysis (TGA) of all three TSILs was carried out at a heating rate of 5 °C min^−1^ from 30 to 600 °C under a nitrogen atmosphere. As shown in Fig. 3a, the TGA curves indicate that these three TSILs exhibit high thermal stabilities and are stable up to 220 °C. The onsets for the thermal decompositions of TSILs **1**-**3** begin at 236, 300 and 224 °C, respectively, and this can be mostly attributed to the unstable structure of the phosphate. The inflections in the TGA curves for TSILs **1**-**3** at 250, 310, and 280 °C, respectively, are due to P-O, P-C and P-N bond cleavages, respectively. Further heating the TSILs to 350~400 °C leads to gradual, nonspecific decomposition. In general, the linking atom between the P-atom and the butyl side chains in the imidazolium influences the thermal stability of the TSILs. Oxygen and nitrogen linking atoms are less stable, and the butyl appendage has the highest temperature for the onset of the thermal decomposition.

Figure 3b shows the influence of the temperature on the densities of the TSILs synthesized in our work. The results indicate that the densities of the TSILs **1**-**3** were approximately 1.361 ± 0.009, 1.301 ± 0.012 and 1.331 ± 0.007 g/mL, respectively, at 25 °C. These densities are close to those of homologous conventional ionic liquids, and the TSILs are promising for the phase separation of immiscible liquid mixtures^40^. The densities of the TSILs at different temperatures are given in Supplementary Table S1.

### Extraction kinetics

**Figure 4:**
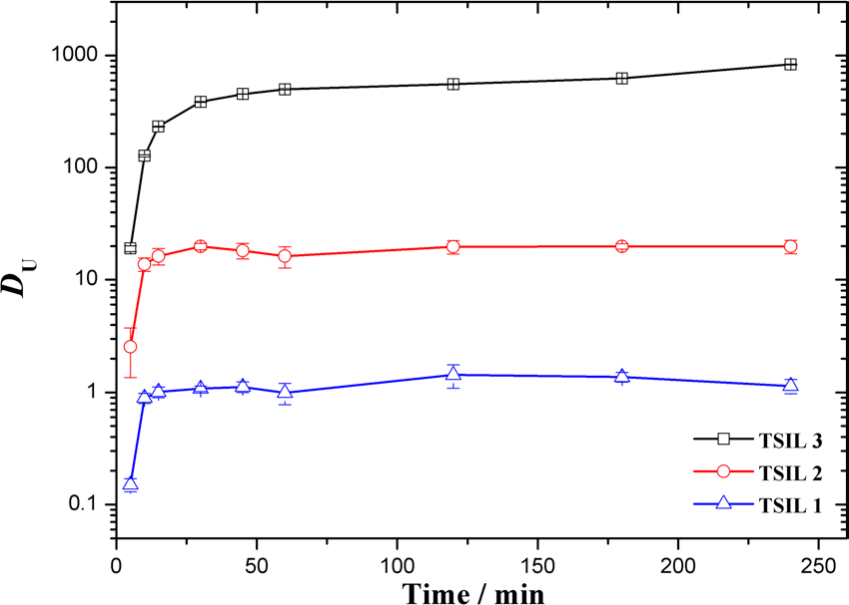
Extraction kinetics of uranyl ions from a 3.0 M HNO_3_ feed into the TSIL/C_4_mimTf_2_N phase.

Studying the extraction kinetics of ionic liquids is important. Due to their high viscosity, the extraction of uranyl ions with an ionic liquid takes a longer time to reach equilibrium compared to extraction with molecular diluents such as *n*-dodecane. Figure 4 compares the extraction profiles of uranyl ions with TSILs as a function of the extraction time. The results show that the relatively slow extraction kinetics follow the order TSIL 3 < TSIL 2≈TSIL 1. TSILs 1 and 2 required 30 min and TSIL 3 required 60 min to reach equilibrium. The observed slow kinetics in these three TSILs agreed with literature reports and can be attributed to the high viscosities of the TSILs^30,31^. In view of the slower mass transfer rates, the subsequent solvent extraction studies were performed with an equilibration time of 2 h to ensure the extraction equilibrium was reached.

### Distribution ratio studies

**Figure 5:**
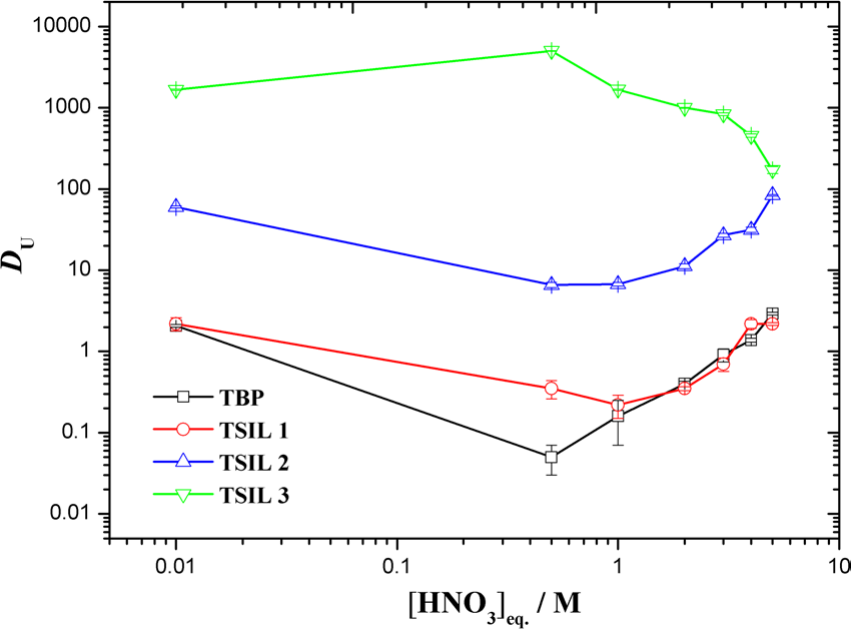
Extraction behavior of uranyl ions from different feed acidities into the TSIL/C_4_mimTf_2_N phase.

TSILs **1, 2** and **3** were used to extract uranyl ions from a nitric acidic solution to compare their uranium extraction efficiencies. For comparison purposes, the distribution data for uranyl ions with TBP in C_4_mimTf_2_N is also presented, and the extraction systems were nearly identical. The variations in the distribution ratio of 50 mg/L of uranyl in TSIL/C_4_mimTf_2_N (1:3 *v*/*v*) as a function of the nitric acid concentration from 0.01 to 5 M are shown in Fig. 5 and Supplementary Table S2.

The extraction of uranyl ions in TSIL **3** had two significant differences compared with that in TSIL **1** and **2** and TBP. First, the distribution ratio of the uranyl ions in TSIL **3** was higher than that in TSIL **1** and **2** and TBP for all the nitric acid concentrations. Contacting the TSIL **3**/C_4_mimTf_2_N phase with a brilliant yellow uranyl nitrate aqueous phase resulted in the yellow color moving into the ionic liquid phase, as shown in Supplementary Fig. S1. The observed extraction efficiency trend for the uranyl ions with the synthesized TSILs was: TSIL **3** > TSIL **2** > TSIL **1** ≈ TBP. A similar trend was observed with phosphoryl-functionalized quaternary ammonium-based ionic liquids^19^.

Second, the uranyl ion extraction trends with the TSILs as the nitric acid concentration changed were different. Typical boomerang-shaped curves were observed for TSIL **1**, TSIL **2** and TBP. As the nitric acid concentration increased, the distribution ratios of the uranyl ions decreased from 2.19 ± 0.40, 59.9 ± 1.7 and 2.07 ± 0.08 at 0.01 M to minimum values of 0.35 ± 0.09, 6.60 ± 0.50 and 0.05 ± 0.02 at 0.05 M and then gradually increased for TSIL **1**, TSIL **2** and TBP, respectively.

In contrast, TSIL **3** demonstrated an entirely different trend in the extraction behavior under the same conditions. As shown in Fig. 5, as the nitric acid concentration increased, the distribution ratios of the uranyl ions increased from 1665 ± 23 at 0.01 M to a maximum value of 4999 ± 51 at 0.05 M, and this was followed by a gradual decrease in the *D* values. These results agreed with literature reports on the extraction of uranyl ions based on a solvation-type mechanism^4,41^.

Visser and Rogers^8^ and Giridhar^11^ *et al*. proposed that uranyl ions can form complex species, such as [UO_2_NO_3_]^+^, UO_2_(NO_3_)_2_, and [UO_2_(NO_3_)_3_]^−^, depending on the nitrate ion concentration. Based on a solvation-type extraction mechanism, the uranyl ions were extracted by TSIL **3** as the neutral UO_2_(NO_3_)_2_ species and involved the following simplified equilibrium:

1 $${{\rm{UO}}}_{2\,{\rm{aq}}}^{2+}+{{\rm{2No}}}_{3\,{\rm{aq}}}^{-}+{\rm{nTSIL}}{3}_{{\rm{IL}}}\iff {{\rm{UO}}}_{2}{({{\rm{NO}}}_{3})}_{2}\cdot {{\rm{nTSIL3}}}_{{\rm{IL}}}$$

where the subscripts ‘aq’ and ‘IL’ refer to the aqueous and ionic liquid phases, respectively. As a result, the initial increase in the *D* values of the uranyl ions with the increasing HNO_3_ concentration at lower acidities is partly due to the increase in the UO_2_(NO_3_)_2_ concentration and partly due to salting out effects, which is in agreement with the observations of Mohapatra *et al*.^4,41^. The decrease in the *D* values of the uranyl ions above 0.05 M HNO_3_ can be explained by two factors. First, the neutral UO_2_(NO_3_)_2_ species is converted to the less extractable anionic [UO_2_(NO_3_)_3_]^−^ species as the HNO_3_ concentration continues to increase. Second, the extraction of H^+^ and NO_3_^−^ increases as the HNO_3_ concentration in the aqueous phase increases, which decreases the effective concentration of TSIL **3** in the ionic liquid phase and results in lower distribution ratios. However, the above extraction mechanism is overly simplified and does not consider the participation of Tf_2_N^−^ and H_2_O, which can also complex with uranyl ions and result in other mechanisms such as ion-exchange. The present data are not sufficient to clearly clarify the mechanism. We expect that our further studies will clarify the coordination chemistry of the uranyl ions in the present ionic liquid systems and the extraction mechanism.

Based on the above observations, we concluded that the linking group between the phosphoryl moiety and the bridging moiety in the cationic segment of the TSILs has an important role in the extraction of uranyl ions. The phosphoramide-containing TSIL had higher extraction efficiencies than the other phosphoryl-functionalized ILs. More importantly, a solvation extraction mechanism was realized in the phosphoramide-containing TSIL-based extraction system, which can reduce the loss or leaching of ILs into the aqueous phase. Therefore, the present data suggested that TSIL **3** is an effective extractant for uranyl ions under a wide range of acidic conditions.

The following studies on the effect of the TSIL **3** concentration and stripping were performed at 3.0 M HNO_3_, which is an acid concentration similar to that encountered in PUREX process streams (2~3 M HNO_3_).

### Extraction stoichiometry

The stoichiometry of the extracted uranyl species with TSIL **3** in C_4_mimTf_2_N was determined by varying the TSIL **3** concentration with a fixed aqueous phase acidity of 3 M HNO_3_. Based on the solvation-type mechanism and Eq. (1), the extraction equilibrium constant (*K*_ex_) for uranyl ion extraction with TSIL **3** can be represented by Eq. (2):

2 $${{K}}_{{\rm{e}}{\rm{x}}}=\frac{{[({{\rm{UO}}}_{2}{({{\rm{NO}}}_{3})}_{2})\cdot {\rm{nTSIL}}3]}_{{\rm{IL}}}}{{[{{\rm{UO}}}_{2}^{2+}]}_{{\rm{aq}}}{[{{\rm{NO}}}_{3}^{-}]}_{{\rm{aq}}}^{2}{[{\rm{TSIL}}3]}_{{\rm{IL}}}^{{\rm{n}}}}$$

**Figure 6:**
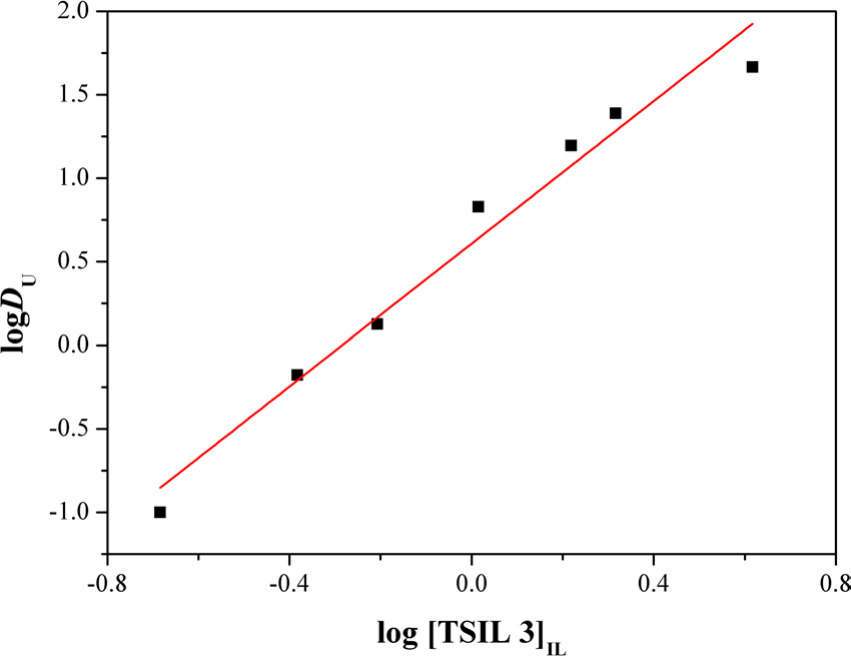
Log–log plot of the distribution ratio for uranyl ions with variable concentrations of TSIL **3** in C_4_mimTf_2_N.

Rearrangement of Eq. (2) results in Eq. (3):

3 $$\mathrm{log}\,{{D}}_{U}={n}\,\mathrm{log}\,{[{\rm{TSIL}}3]}_{{\rm{IL}}}+\,\mathrm{log}\,{{K}}_{{\rm{e}}{\rm{x}}}+2\,\mathrm{log}\,{[{{{\rm{NO}}}_{3}}^{-}]}_{{\rm{aq}}}$$

where *n* represents the extraction stoichiometry. As shown in Fig. 6, the distribution ratio of the uranyl ions increased as the TSIL **3** concentration increased in the ionic liquid phase. A linear fit of the log *D*_U_ vs log [TSIL **3**] plot resulted in a slope value (*n*) of 2.1 ± 0.2, which suggested a 1:2 uranyl ion/TSIL **3** stoichiometry. The species extracted in the present extraction system was UO_2_(NO_3_)_2_(TSIL **3**)_2_.

### Stripping studies

**Figure 7:**
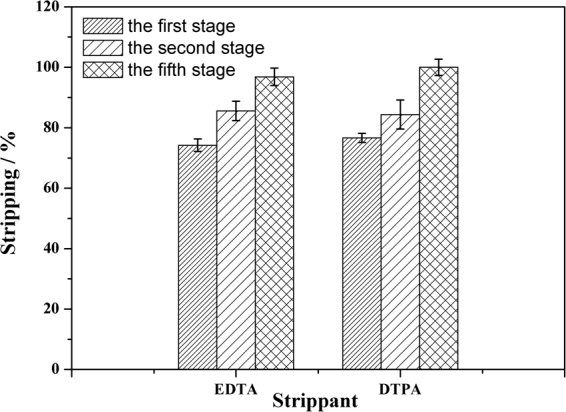
Stripping behavior of uranyl ions from an extract made using TSIL 3/C_4_mimTf_2_N.

Stripping metal ions from the ionic liquid phase is a major challenge. In molecular diluent extraction systems, metal ion extraction is carried out at a higher acidity (3~4 M HNO_3_) with extractants such as TBP or CMPO, and the stripping is performed by contacting the extractant with dilute acid solutions. However, the *D* values of the metal ions are significantly higher at lower acidities in the ionic liquid systems, and quantitative stripping of the metal ions via a suitable adjustment of the acidity of the aqueous phase is not possible. Ethylenediaminetetraacetic acid (EDTA) or diethylenetriamine pentaacetic acid (DTPA) in guanidine carbonate have been previously reported to be effective for quantitative stripping of trivalent lanthanide ions from a CMPO-ionic liquid phase^42,43^. In the present work, 0.05 M EDTA or DTPA in 1.0 M guanidine carbonate was used to strip the uranyl ions from the loaded TSIL **3** extraction system. As shown in Fig. 7, the results suggested that both the EDTA and DTPA solutions in 1.0 M guanidine carbonate could effectively strip ~80% of the uranyl ions in a single stage. The cumulative stripping % values for the uranyl ions were approximately 96.8 ± 2.9% and 99.9 ± 2.7% for EDTA and DTPA, respectively. Therefore, quantitative back extraction of uranyl ions from the ionic liquid phase is possible using a suitable number of stripping stages.

### DFT calculation

The interaction between the uranyl compounds and the TSIL **3** has an essential role in the extraction of the uranyl ions. From a structural point of view, TSIL **1** and TSIL **2** are the same as TSIL **3** except for the linker group between the phosphonium moiety and butyl side chains in the imidazolium cations. Therefore, the phosphoramine functional group in the structure of TSIL **3** may be the main reason for the high uranyl ion extraction efficiency.

To prove the above hypothesis, a DFT computational study was used to investigate the fundamental nature of the interaction between TSIL **3** and uranyl ions. In this sense, TSIL **3** was simplified to **TSILN**, which was used as the simplified mode structure in our computational study (Supplementary Fig. S2c). TBP has been reported to coordinate with uranyl ions via the oxygen atom of the P = O moiety^44^. At the same time, for the neutral UO_2_L_2_(NO_3_)_2_ complexes, the two nitrate anions were coordinated to the U center as bidentate ligands forming a hexagonal bipyramidal structure. Based on the abovementioned references and our experimental and calculation results, two types of coordination modes for the UO_2_(NO_3_)_2_/TSIL complex are illustrated in Supplementary Fig. S2. The first mode was a monodentate coordination mode with a P = O–U interaction (Supplementary Fig. S2a). Two TSIL molecules were proposed to coordinate to one uranium atom via the oxygen atom in the P = O moiety. The second mode was a bidentate chelating mode involving a P-X–U interaction with one TSIL (X = O or N) (Supplementary Fig. S2b).

**Figure 8:**
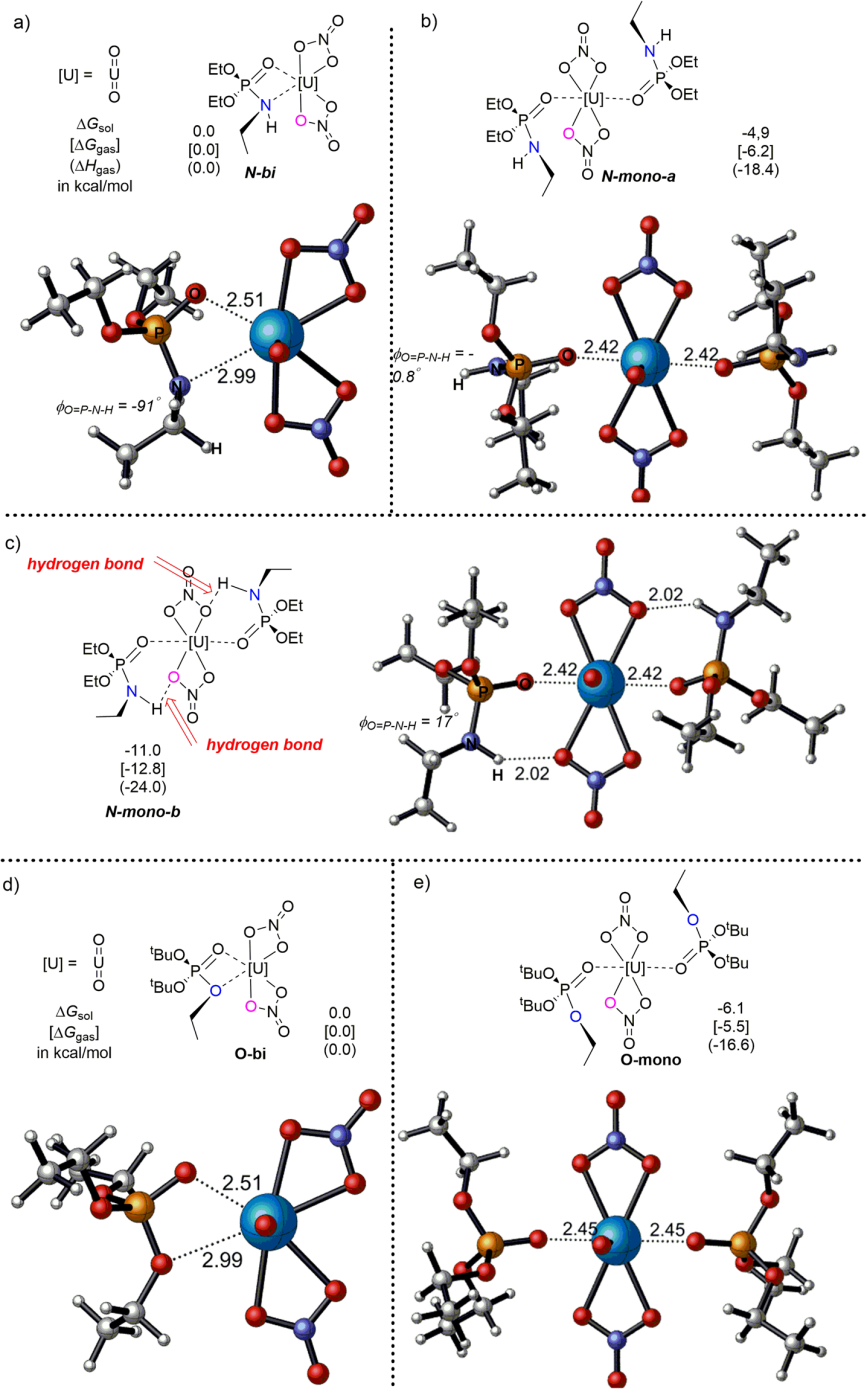
(**a**,**b** and **c**) Relative energies of the uranyl/**TSILN** complexes together with the DFT calculated structures. (**d** and **e**) Relative energies of the uranyl/**TSILO** complexes together with the DFT calculated structures.

The DFT calculations indicated mono- and bi-dentate structures of UO_2_(NO_3_)_2_(**TSILN**)_1or2_, as shown in Fig. 8 (N-**mono-a** and **N-bi**). The calculated data showed that the **N-mono-a** mode was more favored than the **N-bi** structure by 4.9 kcal/mol. The major reason for the relative instability of the **N-bi** structure was its significantly higher enthalpy (18.4 kcal/mol higher) than that of the monodentate structure (**N-mono-a**). In addition, another reason could be the geometry distortion in the phosphoramine moiety. In the optimized **TSILN** structure (Supplementary Fig. S3), O = P-N-H adopts a near plane configuration, which was indicated by the small dihedral angle (27°), and remained in the **N-mono-a** (−0.8°) mode. However, in the **N-bi** structure, the optimal O-P-N-H dihedral angle increased to 91°. Apparently, the N atom distorted into a tetrahedral configuration to coordination with the U center, which increased the energy of the bidentate structure. Therefore, the monodentate structure, UO_2_(NO_3_)_2_(**TSIL 3**)_2_, was more favorable than the bidentate structure, UO_2_(NO_3_)_2_(**TSIL 3**)_1_. These theoretical results revealed the coordination mode of the UO_2_(NO_3_)_2_/TSIL 3 complex and supported the extraction stoichiometry experimental data, which indicated a stoichiometry of 2 (Fig. 6).

Based on the monodentate model **N-mono-a** structure above, an alternative monodentate model structure, **N-mono-b**, was located (Fig. 8c), which was 6.1 kcal/mol more favorable than **N-mono-a** in terms of the Gibbs free energy in water. In **N-mono-b**, the N-H attached to the phosphonium group in **TSILN** is hydrogen bonded to the O atom in the nitrite coordinated to the U center, which was demonstrated by the short distances of the N-H–O bonds (1.99 and 1.98 Å). The hydrogen bond between TSIL **3** and uranyl nitrate further stabilized the UO_2_(NO_3_)_2_(TSIL **3**)_2_ complex. Furthermore, DFT calculation results (Supplementary Table S3) also showed the binding energies of the neutral UO_2_(NO_3_)_2_(TSILN)_2_ complexes were more negative than those of the charged UO_2_(NO_3_)(TSILN)_2_^+^ and UO_2_(NO_3_)_3_(TSILN)_2_^−^ complexes. Therefore, two interactive sites exist within the structure of TSIL **3**, the P = O functional group of the phosphoryl moiety and the amine group (NH) connected to the phosphoryl moiety.

To further confirm the important role of the phosphoramine functional group in the TSIL **3** structure, the fundamental nature of the interaction between uranyl nitrate and TSIL **1** (simplified as **TSILO** in Supplementary Fig. S2c) was investigated, and the calculation results were compared with those for TSIL **3**. Two possible types of interactions including mono- or bi-dentate structures of UO_2_(NO_3_)_2_(**TSILO**)_1or2_ were determined and are illustrated in Fig. 8c and d (O-**mono** and **O-bi**). Similar to the UO_2_(NO_3_)_2_/TSIL **3** complex, the **O-mono** mode was 6.1 kcal/mol more favorable than the **O-bi** structure. However, TSIL **2** cannot form a hydrogen bond with uranyl nitrate since the ether connected to the phosphonium group does not have a hydrogen. Therefore, the phosphoramine functional group in TSIL **3** was the major reason for the high uranyl ion extraction efficiency. Further computational studies using the full models of the TSILs are ongoing. Combined with more experimental evidence, these studies will allow an in-depth understanding of the coordination behavior between TSILs and uranyl nitrate.

## Conclusions

In this work, a class of TSILs with phosphoryl groups incorporated into the butyl bridging moieties of the imidazolium cations with different linking groups was synthesized and characterized. The influence of the linking group in the TSILs on uranyl ion extraction efficiencies was comprehensively investigated experimentally and theoretically. The synergistic effect of coordination and hydrogen bonding led to dramatically higher *D* values for uranyl ions with the phosphoramidate-based TSILs compared with those for other phosphoryl-based TSILs. A solvation extraction mechanism can be used for the phosphoramide-containing TSIL-based extraction system. Stripping the uranyl ions from the TSILs was possible using a solvent system with buffered complexing agents, such as EDTA or DTPA. The results of this study demonstrate the importance of hydrogen bonding in the chemical structure of the extractants and support the idea of rational design, synthesis and applications of TSILs for uranyl ion extraction.

## Methods

### Materials and instrumentation

All the chemicals used in this study were analytical grade. 1-Butyl-3-methylimidazolium bis(trifluoromethanesulfonyl)imide (C_4_mimTf_2_N) was procured from Shanghai Cheng Jie Chemical Co., LTD., China. *N*-Methylimidazole, 4-bromo-1-butanol, dibutyl phosphate, lithium bis(trifluoromethanesulfonyl)imide (LiTf_2_N), and other reagents were purchased from Aladdin Industrial Corporation. Nuclear magnetic resonance (NMR) spectra were recorded on a Bruker Avance 400 MHz spectrometer. TGA was conducted on a TA 2950 instrument. The densities were determined using an Anton Paar DMA 5000 M oscillating tube density meter. The concentration of uranium was measured using an AttoM (Nu Instruments, Wreham, UK) high-resolution sector field inductively coupled plasma mass spectrometer (SF-ICP-MS).

### Synthesis of 3-(4-((dibutoxyphosphoryl)oxy)butyl)-1-methyl-imidazole bis((trifluorometyhyl)sulfonyl)amide (TSIL 1)

The synthesis procedure was similar to the reported method with some modifications^22^. A solution of dibutyl phosphate (0.25 mol) in CCl_4_ (60 ml) was added dropwise to a stirred solution of 50% aqueous sodium hydroxide (60 ml), 1,4 butanediol (0.2 mol), carbon tetrachloride (CCl_4_, 50 ml), methylene chloride (DCM, 50 ml) and a phase-transfer catalyst, tetrabutylammonium bromide (TBAB, 0.5 mmol). Then, the mixture was stirred for 3 h at 25 °C. The mixture was diluted with DCM (50 ml) and filtered, and the organic layer was washed with 2% aqueous hydrogen chloride (25 ml) and water (2 × 25 ml). The layer was then dried with anhydrous sodium sulfate, and the solvents were distilled under reduced pressure. Compound **2** was obtained as a colorless liquid. TBAB (1.2 mmol) was added at 25 °C to a flask containing a stirring mixture of 1,2-dichloro-4,5-dicyanobenzoquinone (DDQ, 1.2 mmol), triphenylphosphine (PPh_3_, 1.2 mmol) in dry DCM (250 ml). Then, compound **2** (1.0 mmol) was added to the mixture. The reaction was complete in 20 min to obtain a pale yellow liquid, compound **3**. The crude compound **3** and 1-methylimidazol were added to a round-bottomed flask in a mole ratio of 1.2:1 and refluxed by heating and stirring at 70 °C for 72 h. The resulting viscous product, compound **5**, was allowed to cool to room temperature and then washed three times with 200 ml portions of ethyl acetate (EA). After the last washing, the remaining EA was removed under vacuum. A solution of LiTf_2_N (30 mmol) in deionized water was added to the aqueous solution of compound **5** (20 mmol), and the suspension solution was stirred vigorously for 20 h at 25 °C. The top aqueous layer was removed, and the ionic liquid was washed with deionized water and dried at 70 °C in vacuum to afford TSIL **1** (6.24 g, 90.2%). ^1^H NMR (Supplementary Fig. S4) (400 MHz, CDCl_3_): *δ* = 0.90 (t, 6 H *J* = 7.2 Hz, OCH_2_CH_2_CH_2_CH_3_), 1.36 (m, 4 H, OCH_2_CH_2_CH_2_CH_3_), 1.62 (m, 4 H, NCH_2_CH_2_CH_2_CH_2_OPO(OCH_2_CH_2_CH_2_CH_3_)_2_), 1.70 (m, 2 H, NCH_2_CH_2_CH_2_CH_2_OPO(OCH_2_CH_2_CH_2_CH_3_)_2_), 1.97 (m, 2 H, OCH_2_CH_2_CH_2_CH_3_), 3.89 (s, 3 H, NCH_3_), 4.00 (m, 6 H, NCH_2_CH_2_CH_2_CH_2_OPO(OCH_2_CH_2_CH_2_CH_3_)_2_), 4.21 (t, 2 H, *J* = 7.0 Hz, NCH_2_CH_2_CH_2_CH_2_), 7.32 (s, 1 H, NCHCHN), 7.39 (s, 1 H, NCHCHN), 8.73 (s, 1 H, NCHN) ppm. ^13^C NMR (Supplementary Fig. S5) (126 MHz, CDCl_3_): *δ* = 135.91, 124.65, 122.57, 119.7 (q, *J* = 322 Hz), 67.7 (d, *J* = 6.3 Hz), 66.28 (d, *J* = 6.3 Hz), 49.37, 36.27 (d, *J* = 11.3 Hz), 32.19 (d, *J* = 7.6 Hz), 26.61 (d, *J* = 7.6), 26.41, 18.56, 13.42 ppm. ^19^F NMR (Supplementary Fig. S6) (470 MHz, CDCl_3_): *δ* = −79.20 ppm. ^31^P NMR (Supplementary Fig. S7) (162 MHz, CDCl_3_): *δ* = −1.17 ppm. LC-MS (ESI) m/z (%): calcd for C_16_H_32_N_2_O_4_P (M^+^) 347.21, found 347.17.

### Synthesis of 3-(5-(dibutoxyphosphoryl)pentyl)-1-methyl-imidazolbis((trifluoro methyl) sulfonyl)amide (TSIL 2)

A solution of dibutyl phosphate (10.3 mmol) in THF (10 ml) was added dropwise to a stirred aqueous solution of 60% sodium hydride (10 mmol) and tetrahydrofuran (THF, 20 ml) in a 100 ml round bottom flask. The mixture was heated to reflux and maintained under reflux conditions for 1 h. Then, a solution of 1,5-dibromopentane (14.5 mmol) in THF (10 ml) was slowly added, and the mixture was refluxed for 12 h. The product was extracted into EA (3 × 15 ml). The EA layer was dried with anhydrous sodium sulfate and concentrated under reduced pressure, and the residue was dried under vacuum for 4 h to afford compound **4** as a colorless oil in a quantitative yield. A solution of 1-methylimidazol (2.5 mmol) in DCM (5 ml) was added to a solution of compound **4** (3.5 mmol) in DCM (10 ml), and the mixture was stirred at 45 °C for 72 h. Then, the solvent was evaporated under reduced pressure and washed three times with 20 ml portions of EA. Afterwards, the remaining EA was removed under vacuum to obtain compound **6**. LiNTf_2_ (3 mmol) was added to a solution of compound **6** (2.5 mmol) in water and DCM (2 ml/2 ml), and the mixture was stirred at 25 °C overnight. Then, the mixture was washed with deionized water (3 × 5 ml). The DCM layer was dried with anhydrous sodium sulfate and concentrated under reduced pressure, and the residue was dried under vacuum overnight to afford the viscous TSIL **2** (0.76 g, 89.1%). ^1^H NMR (Supplementary Fig. S8) (400 MHz, CDCl_3_): *δ* = 0.93 (t, *J* = 4.0 Hz, 6 H, *CH*_3_(CH_2_)3°), 1.38 (m, 4 H, CH_3_ *CH*_2_(CH_2_)2°), 1.45 (m, 2 H, NCH_2_ *CH*_2_CH_2_CH_2_CH_2_PO(OCH_2_CH_2_CH_2_CH_3_)_2_), 1.63 (m, 6 H, CH_3_CH_2_ *CH*_2_CH_2_O, NCH_2_CH_2_ *CH*_2_CH_2_CH_2_PO(OCH_2_CH_2_CH_2_CH_3_)_2_), 1.74 (m, 2 H, NCH_2_CH_2_CH_2_ *CH*_2_CH_2_PO(OCH_2_CH_2_CH_2_CH_3_)_2_), 1.89 (m, 2 H, NCH_2_CH_2_CH_2_CH_2_ *CH*_2_PO(OCH_2_CH_2_CH_2_CH_3_)_2_), 3.96 (m, 3 H, N*CH*_3_), 4.01 (m, 4 H, CH_3_CH_2_CH_2_ *CH*_2_O), 4.18 (t, *J* = 4.0 Hz, 2 H, N*CH*_2_CH_2_CH_2_CH_2_ CH_2_PO(OCH_2_CH_2_CH_2_CH_3_)_2_), 7.31 (s, 1 H, N*CHCH*N), 7.34 (s, 1 H, N*CHCH*N), 8.84 (s, 1 H, N*CH*N) ppm. ^13^C NMR (Supplementary Fig. S9) (126 MHz, CDCl_3_): *δ* = 136.30, 123.60, 122.30, 119.8 (q, *J* = 322 Hz), 65.39 (d, 7.6 Hz), 49.83, 36.35, 32.54, 32.49, 29.51, 26.71 (d, 15.1 Hz), 25.31, 24.19, 21.80 (d, 5.0 Hz), 18.70, 13.55 ppm. ^19^F NMR (Supplementary Fig. S10) (470 MHz, CDCl_3_): δ = −79.03 ppm. ^31^P NMR (Supplementary Fig. S11) (162 MHz, CDCl_3_): *δ* = 31.39 ppm. LC-MS (ESI) m/z (%): calcd for C_17_H_34_N_2_O_3_P (M^+^) 345.23, found 345.35.

### Synthesis of 3-(4-((dibutoxyphosphoryl)amino)butyl)-1-methyl-imidazolbis ((trifluoro methyl) sulfonyl)amide (TSIL 3)

A solution of dibutyl phosphorochloridate (0.1 mol) in DCM (50 ml) was added dropwise to a stirred solution of 1-(4-aminobutyl)imidazole (0.1 mol), triethylamine (TEA, 0.3 mol), and dichloromethane (50 ml). The mixture was stirred overnight at 25 °C. Then, the mixture was filtered, and the organic layer was distilled under reduced pressure to obtained compound **7**. In the next step, iodomethane (0.1 mol) in ether (30 ml) was added to a flask containing a stirred solution of compound **7** (0.1 mol) in ether (30 ml). The mixture was stirred overnight at 25 °C. Then, the lower layer was collected and washed 3 times with ether. After the last washing, the remaining ether was removed under vacuum to obtain the viscous product, compound **8**. A solution of LiNTf_2_ (0.15 mol) in deionized water (30 ml)was added to an aqueous solution of compound **8** (0.1 mol, 30 ml), and the mixture was stirred at 25 °C for 24 h. Afterwards, the top aqueous layer was removed, and the ionic liquid layer was washed with deionized water at least 3 times and dried at 70 °C under a vacuum to afford TSIL **3** (28.9, 81.2%). ^1^H NMR (Supplementary Fig. S12) (400 MHz, CDCl_3_): δ = 0.90 (t, *J* = 5.6 Hz, 6 H, *CH*_3_(CH_2_)3°), 1.36 (m, 4 H, CH_3_ *CH*_2_(CH_2_)2°), 1.51 (m, 2 H, NCH_2_ *CH*_2_CH_2_CH_2_NHPO(OCH_2_CH_2_CH_2_CH_3_)_2_), 1.58 (m, 4 H, CH_3_CH_2_ *CH*_2_CH_2_O), 1.91 (m, 2 H, NCH_2_CH_2_ *CH*_2_CH_2_NHPO(OCH_2_CH_2_CH_2_CH_3_)_2_), 2.92 (m, 2 H, NCH_2_CH_2_CH_2_ *CH*_2_NHPO(OCH_2_CH_2_CH_2_CH_3_)_2_), 3.12 (m, 1 H, NCH_2_CH_2_CH_2_CH_2_N*H*PO(OCH_2_CH_2_CH_2_CH_3_)_2_), 3.89 (s, 3 H, NC*H*_3_), 3.92 (m, 4 H, CH_3_CH_2_CH_2_ *CH*_2_O), 4.18 (t, *J* = 6.0 Hz, 2 H, N*CH*_2_CH_2_CH_2_CH_2_NHPO(OCH_2_CH_2_CH_2_CH_3_)_2_), 7.31 (s, 1 H, NCHCHN), 7.40 (s, 1 H, NCHCHN), 8.78 (s, 1 H, NCHN) ppm. ^13^C NMR (Supplementary Fig. S13) (126 MHz, CDCl_3_): δ = 136.07, 123.64, 122.46, 119.73 (q, *J* = 322 Hz), 66.19 (d, *J* = 5.0 Hz), 49.46, 40.18, 36.19, 32.28 (d, *J* = 6.3 Hz), 27.90, 27.88 (d, *J* = 5.0 Hz), 18.70, 13.50 ppm. ^19^F NMR (Supplementary Fig. S14) (470 MHz, CDCl_3_): δ = −79.12 ppm. ^31^P NMR (Supplementary Fig. S15) (162 MHz, CDCl_3_): *δ* = 9.10 ppm. LC-MS (ESI) m/z (%): calcd for C_16_H_33_N_3_O_3_P (M^+^) 346.23, found 346.35.

All three TSILs were subjected to standard silver-ion tests to ensure the complete exchange of the halide for the NTf_2_^−^ anion.

### Distribution ratio studies

Extraction studies were performed by equilibrating equal volumes (usually 1 ml) of the ionic liquid phase with an aqueous solution containing 50 mg/L of uranyl ions at a given nitric acid concentration for 2 h with shaking at 250 rpm at 25 °C. The ionic liquid phases were prepared by diluting the TSILs into C_4_mimTf_2_N at a 1:3 volume ratio. The feed solution was prepared by dissolving a certain amount of uranyl nitrate salt in aqueous nitric acid to obtain the required concentration. After the extraction process reached equilibrium, the phases were separated by centrifugation, and the concentrations of the uranyl ions in the aqueous phase were measured by SF-ICP-MS. The concentration of the uranyl ions extracted into the IL phase was calculated by mass balance. All the results were reproducible and were within an error limit of ±5% error.

The reaction kinetics were studied by shaking equal volumes of the ionic liquid phase and the feed acid solution containing 50 mg/L uranyl nitrate for different times ranging from 15 to 300 min.

The effect of the nitric acid concentration on the uranyl ion extraction was studied by mixing 1 ml of the ionic liquid phase with an equal volume of the aqueous phase containing 50 mg/L uranyl nitrate at nitric acid concentrations ranging from 0.01 to 5 M.

The effect of the TSIL **3** concentration on the uranyl ion extraction in the ionic liquid was studied by equilibrating 1 ml of the ionic liquid phase with an equal volume of a 3 M aqueous nitric acid phase containing 50 mg/L uranyl nitrate. The concentration of TSIL **3** in the ionic liquid phase was varied from 0.05 M to 0.5 M.

The distribution ratio (*D*) and extraction efficiency (*E*%) with equal volumes of the aqueous and ionic liquid phases were calculated according to the following equations:

4 $${D}=\frac{{{C}}_{{\rm{I}}{\rm{L}}}}{{{C}}_{{\rm{a}}{\rm{q}}}}$$

5 $${E} \% =\frac{{C}_{{\rm{IL}}}}{{C}_{{\rm{IL}}}+{C}_{{\rm{aq}}}}\times 100$$

where *C*_IL_ and *C*_aq_ are the uranyl ion concentrations in the ionic liquid phase after the extraction and the aqueous phase after the extraction, respectively.

### Stripping studies

A loaded ionic liquid phase containing uranyl ions after the extraction was employed for the stripping tests. The stripping solution was either 0.05 M DTPA or EDTA in 1.0 M guanidine carbonate. After the stripping, the uranyl ion concentration in the stripping phase was measured. The stripping percentage (%*S*) can be expressed by the following equation:

6 $${S} \% =\frac{{C}_{{\rm{IL}},0}-{C}_{\text{aq},{\rm{s}}}}{{C}_{IL,0}}\times 100$$

where *C*_IL,0_ is the uranyl ion concentration in the ionic liquid phase before the stripping process, and *C*_aq,s_ is the uranyl ion concentration in the stripping phase after the stripping process.

### DFT calculations

All the calculations were carried out using Gaussian09^45^. Geometric optimizations of all the species were carried out using the B3LYP functional^46,47^. The 6–31 G(d) basis set was used for all the atoms, expect for uranium, which was described by the SDD basis set^48^. This method has been successfully used in several reports and has been demonstrated to generate computational results for uranyl complex energies that are in good agreement with experimental data^49,50^. Solvation corrections on the free energies were performed using the CPCM^51^ dielectric continuum solvent model for water employing the UFF radii for all the elements. The solvation calculations were carried out on gas-phase optimized structures under the 6–311 + G(d,p) basis set. Unless otherwise specified, all of the energies discussed in the paper are Gibbs energies at 298 K in a water solution (ΔG_sol_). The Gibbs free energies in the gas phase at 298 K (ΔG_gas_) and the gas-phase enthalpies (ΔH_gas_) are also provided for reference.

## Electronic supplementary material

Supplementary Information
