# Supplementary material for: Significant enhanced uranyl ions extraction efficiency with phosphoramidate-functionalized ionic liquids via synergistic effect of coordination and hydrogen bond
Source: Sci Rep. 2017 Nov 16;7:15735. doi: 10.1038/s41598-017-15899-0 (PMC5691085; doi:10.1038/s41598-017-15899-0)
Supplement: Supplementary file 1 — Supplementary Information [file 41598_2017_15899_MOESM1_ESM.doc]

**Supporting Information**

**Significant enhanced uranyl ions extraction efficiency with phosphoramidate-functionalized ionic liquids via synergistic effect of coordination and hydrogen bond**

Xiang Xie^1^, Zhen Qin^2^, Yao He^1^, Penghui Xiong^2^, Zeng Huang^1^, Yiwu Mao^1^, Hongyuan Wei^1^ & Liangang Zhuo^1*^

Table of Contents

1. **Densities of TSILs at different temperature-----------------------------------------------------S2**
2. **Distribution ratios of uranyl ions in various extraction systems---------------------------------------S3**
3. **Extraction of uranyl ions with TSIL 3 as the extractant in the C_4_mimTf_2_N-------------------------S4**
4. **Coordination modes of uranyl/TSILs complex------------------------------------------------------------S5**
5. **Additional Calculation Results--------------------------------------------------------------------------------S6**
6. **Relative Energies (kcal/mol) of the uranyl/TSILN complexes------------------------------------------S7**
7. **NMR Spectra -----------------------------------------------------------------------------------------------------S8**

**^1^Institute of Nuclear Physics and Chemistry, China Academy of Engineering Physics, Mianyang, 621900, China.**

**^2^Institute of Materials, China Academy of Engineering Physics, Mianyang, 621900, China.**

***Corresponding authors. Tel: +86 816 2485481.**

**E-mail addresses: zhuoliangang2012@163.com (L. G. Zhuo).**

**1. Densities of TSILs at different temperatures.**

| **Temperature**  **(℃)** | **Density**  **(g/mL)** | | |
| --- | --- | --- | --- |
|  | TSIL 1 | TSIL 2 | TSIL 3 |
| **25** | 1.361 ± 0.009 | 1.301 ± 0.012 | 1.331 ± 0.007 |
| **30** | 1.357 ± 0.005 | 1.297 ± 0.008 | 1.327 ± 0.01 |
| **35** | 1.352 ± 0.01 | 1.293 ± 0.005 | 1.323 ± 0.007 |
| **40** | 1.348 ± 0.007 | 1.288 ± 0.009 | 1.318 ± 0.013 |
| **45** | 1.344 ± 0.006 | 1.284 ± 0.003 | 1.314 ± 0.008 |
| **50** | 1.339 ± 0.011 | 1.279 ± 0.011 | 1.310 ± 0.009 |

**Table S1** Temperature dependence of density data for the TSILs.

**2**. Distribution ratios of uranyl ions in various extraction systems

| **[HNO_3_]/M** | **Distribution ratios** | | | |
| --- | --- | --- | --- | --- |
|  | TSIL **1/**C_4_mimTf_2_N | TSIL **2/**C_4_mimTf_2_N | TSIL **3/**C_4_mimTf_2_N | TBP**/**C_4_mimTf_2_N |
| **0.01** | 2.19 ± 0.40 | 59.9 ± 1.7 | 1665 ± 23 | 2.07±0.08 |
| **0.5** | 0.35 ± 0.09 | 6.60 ± 0.50 | 4999 ± 51 | 0.05±0.02 |
| **1** | 0.22 ± 0.07 | 6.74 ± 0.35 | 1666 ± 26 | 0.16±0.09 |
| **2** | 0.35 ± 0.02 | 11.2 ± 0.8 | 999 ± 12 | 0.40±0.03 |
| **3** | 0.70 ± 0.13 | 26.9 ± 1.6 | 823 ± 34 | 0.91±0.17 |
| **4** | 2.19 ± 0.27 | 31.5 ± 2.3 | 453 ± 21 | 1.38±0.08 |
| **5** | 2.18 ± 0.09 | 83.7 ± 1.8 | 171 ± 17 | 2.92±0.11 |

**Table S2** Extraction of uranyl ions using various extraction systems from nitric acid feeds with different HNO_3_ concentrations. The ionic liquid phases were prepared by diluted the TSILs or TBP into C_4_mimTf_2_N with 1:3 volume ratio.

**3. Extraction of uranyl ions with TSIL 3 as the extractant in the C_4_mimTf_2_N**

**
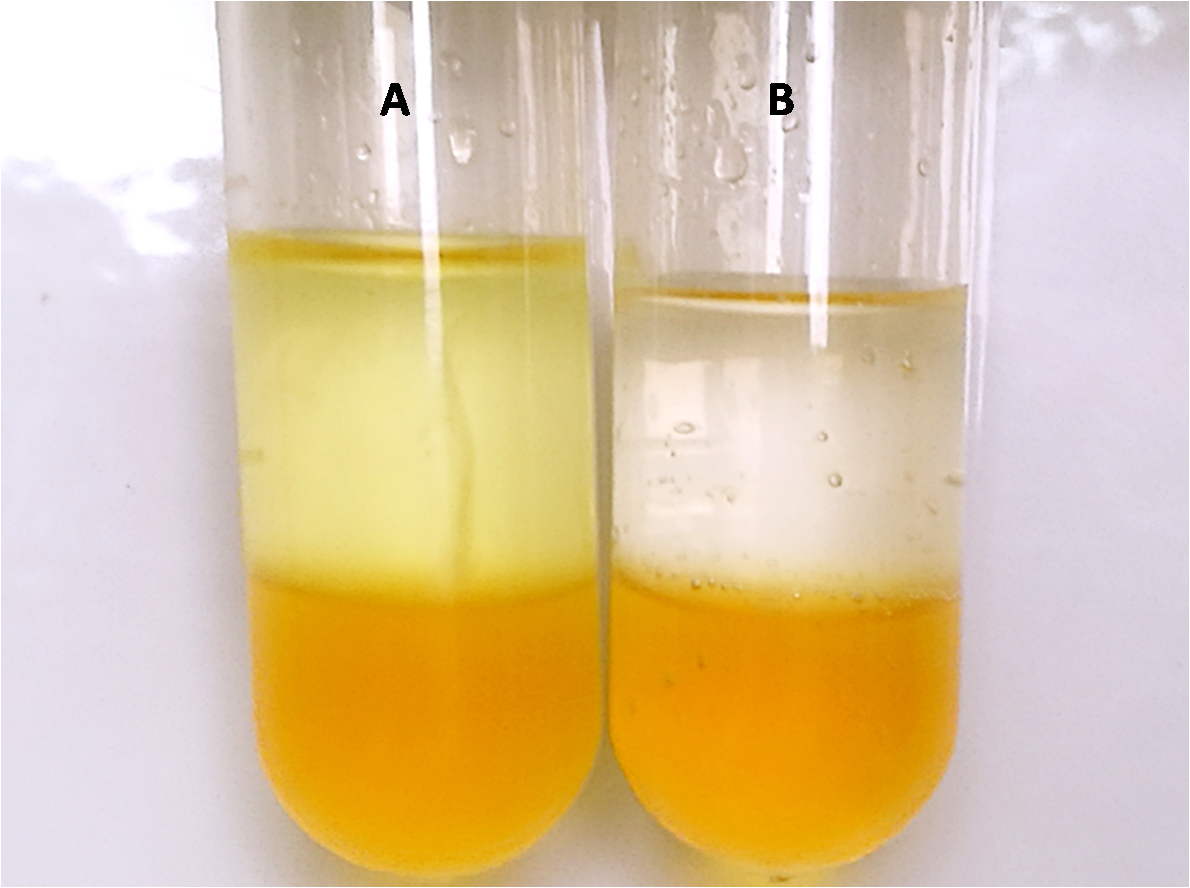
**

**Figure S1.** Extraction of uranyl ions with TSIL **3** as the extractant in the C_4_mimTf_2_N. A: initial solution containing 50 mg/L in 3.0 M HNO_3_ contacted with ionic liquid phase. B: same tube after extraction. The aqueous phase is the upper layer and the ionic liquid phase is the lower layer.

**4. Coordination modes of uranyl/TSILs complex**

**Figure S2** (a) and (b) Possible coordination modes of uranyl/TSILs complex. (c) Simplified models of TSILs for the theoretical study.

**5. Additional Calculation Results**

**Figure S3** The optimized structure of TSILN.

6. **Relative Energies (kcal/mol) of the uranyl/TSILN complexes**

| Species | ΔGg | ΔGsol |
| --- | --- | --- |
| UO_2_(NO_3_)_2_(TSILN)_2_^a^ | 0.0 | 0.0 |
| UO_2_(NO_3_)(TSILN)_2_^+^ | 112.2 | 4.3 |
| UO_2_(NO_3_)(H_2_O)(TSILN)_2_^+^ | 100.0 | 6.2 |
| UO_2_(NO_3_)(H_2_O)_2_(TSILN)_2_ | 94.2 | 13.6 |
| UO_2_(NO_3_)_3_(TSILN)_2_^-^ | -7.8 | 22.7 |

^a^ TSILN is the structure of N-mono-b (Figure 8c)

**Table S3 Relative Energies (kcal/mol) of the uranyl/TSILN complexes.**

**7. NMR Spectra**

**7.1 3-(4-((dibutoxyphosphoryl)oxy)butyl)-1-methyl-imidazolebis((trifluorometyhyl)sulfonyl) amide (TSIL 1):**


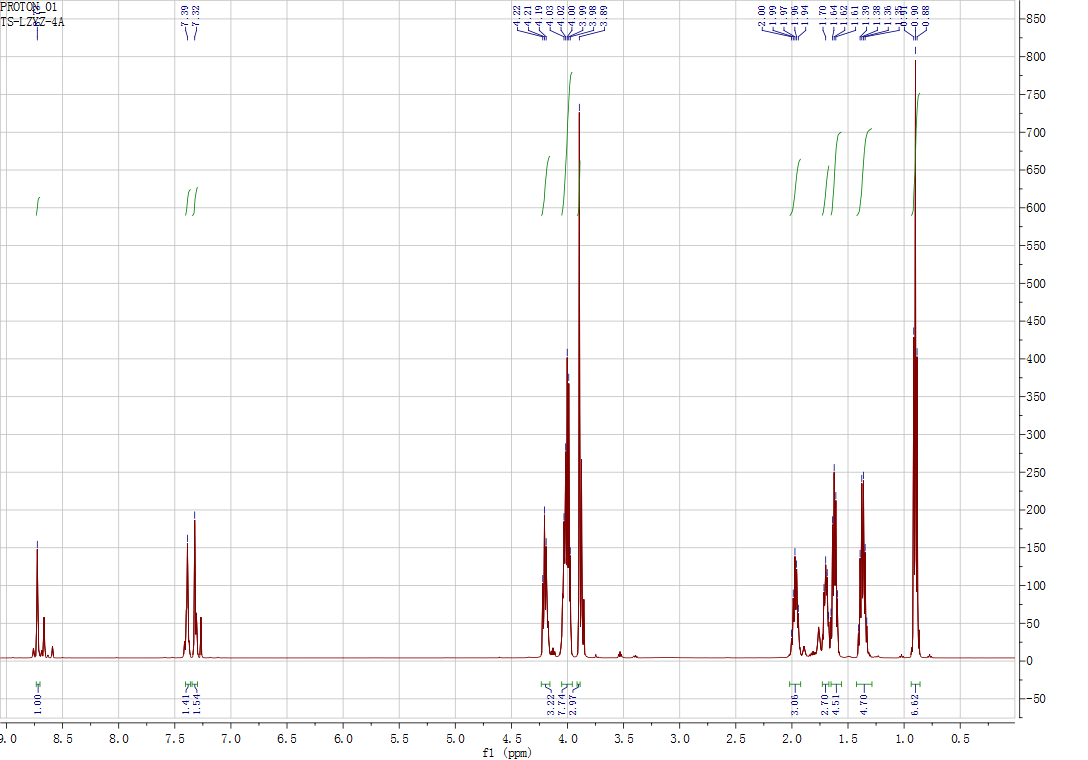


**Figure S4.** ^1^H NMR spectrum of TSIL **1**.


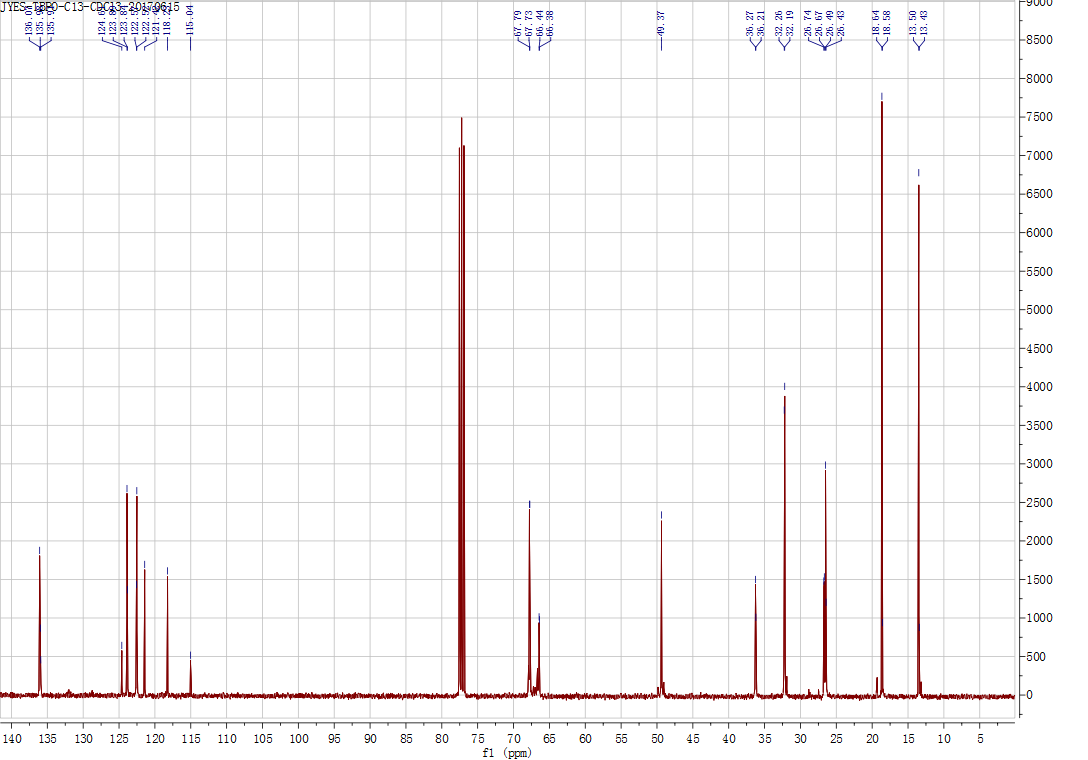


**Figure S5.** ^13^C NMR spectrum of TSIL **1**.


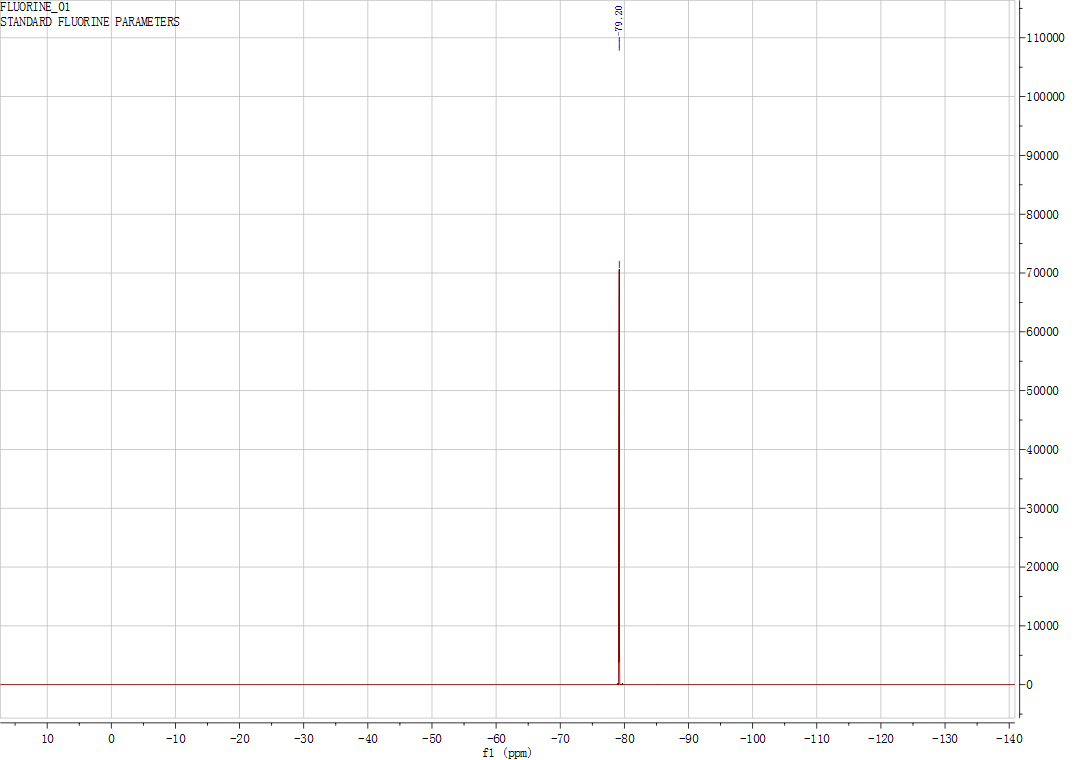


**Figure S6.** ^19^F NMR spectrum of TSIL **1**.


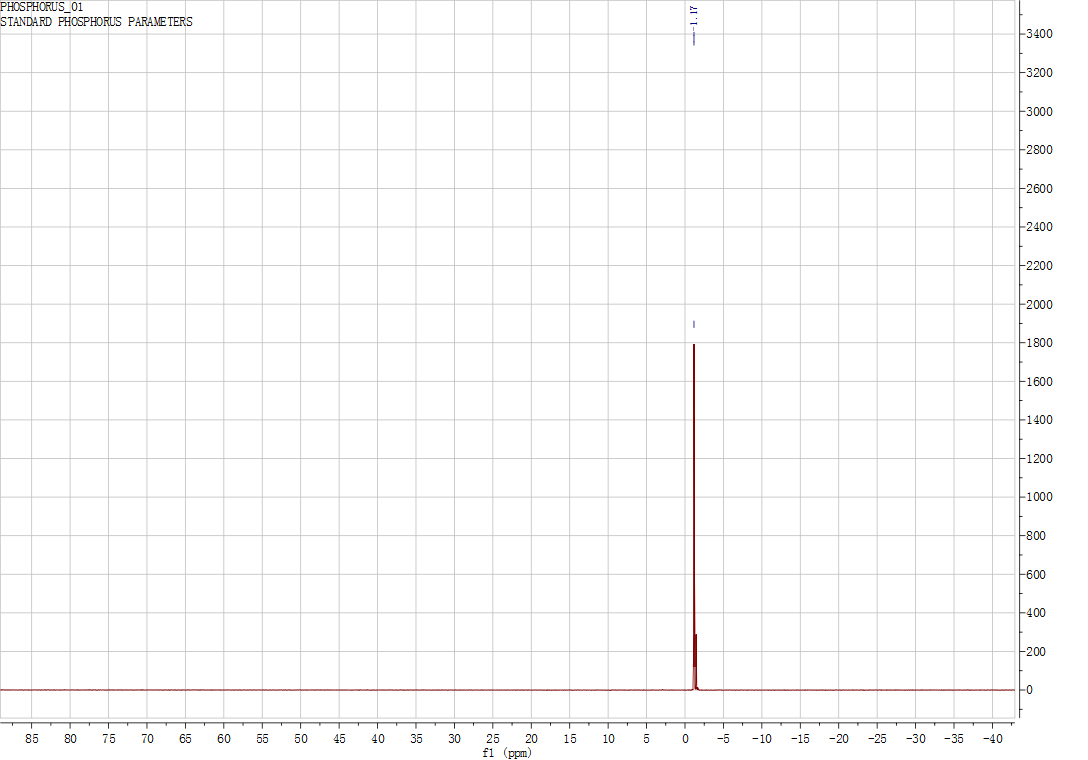


**Figure S7.** ^31^P NMR spectrum of TSIL **1**.

**7.2 3-(5-(dibutoxyphosphoryl)pentyl)-1-methyl-imidazolbis((trifluoromethyl)sulfonyl) amide (TSIL 2)：**


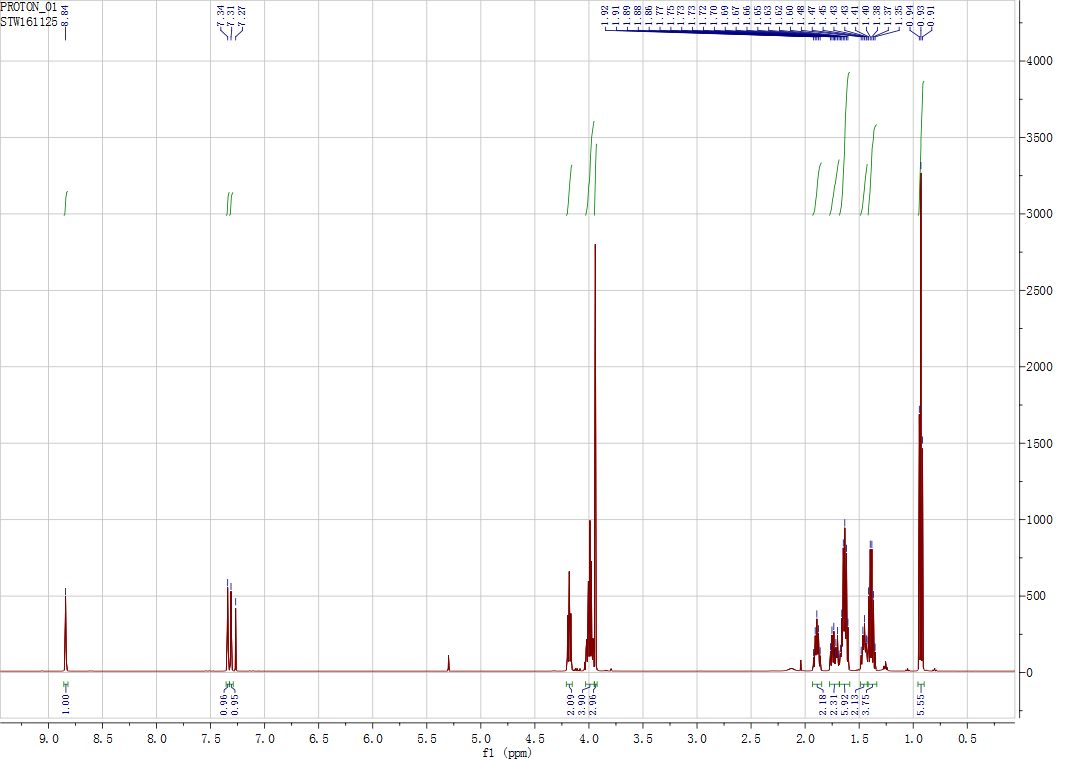


**Figure S8.** ^1^H NMR spectrum of TSIL **2**.


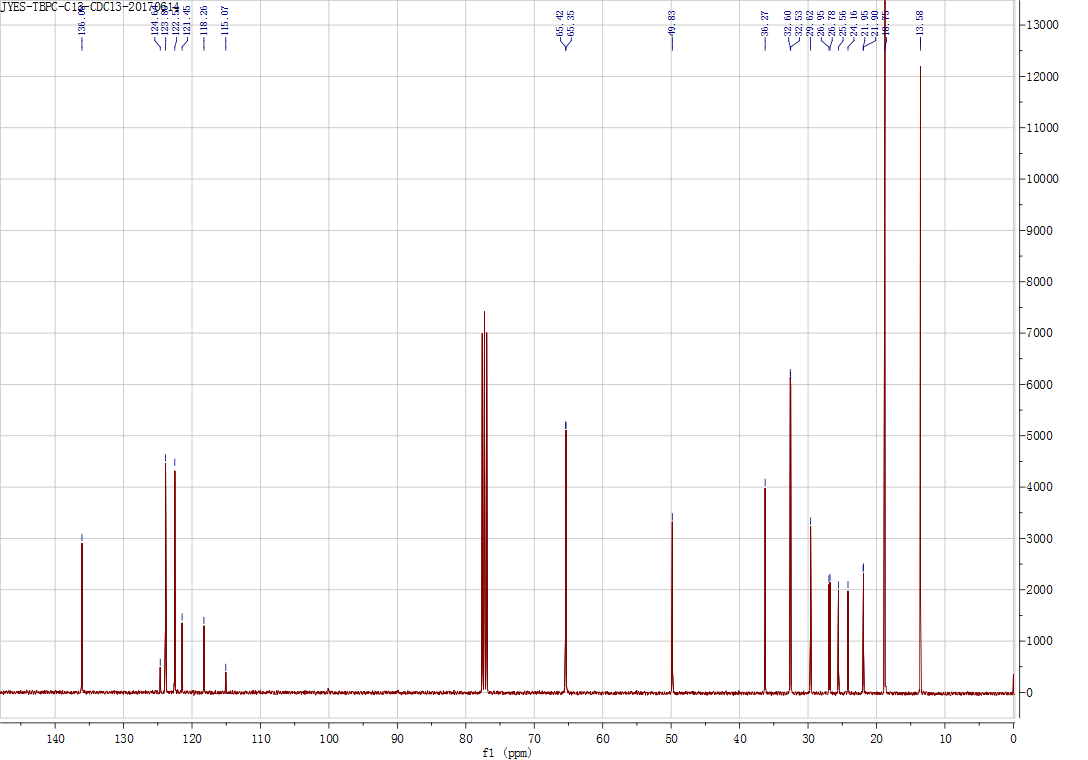


**Figure S9.** ^13^C NMR spectrum of TSIL 2.


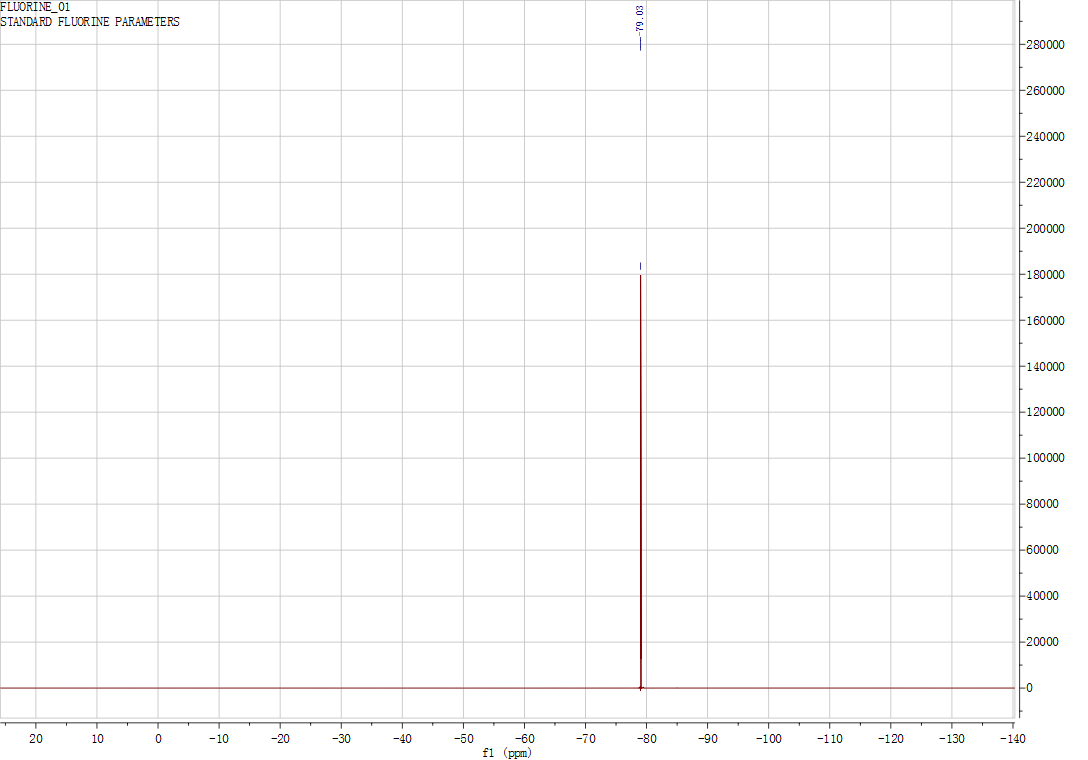


**Figure S10.** ^19^F NMR spectrum of TSIL 2.


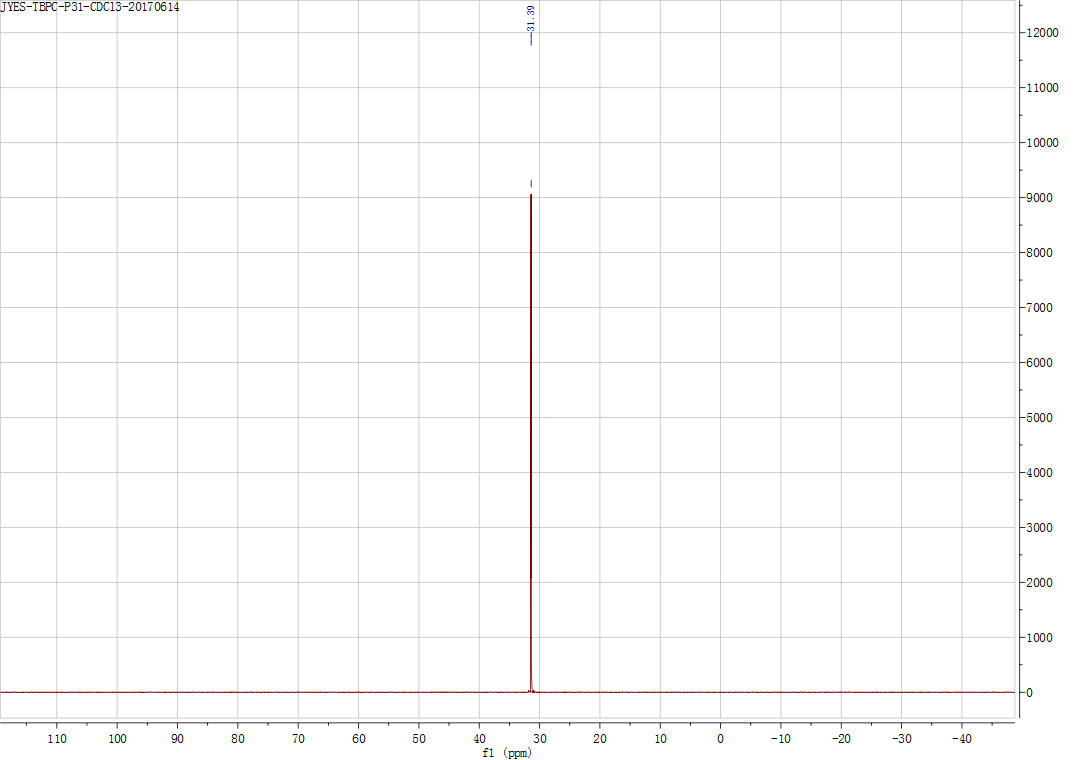


**Figure S11.** ^31^P NMR spectrum of TSIL 2.

**7.3 3-(4-((dibutoxyphosphoryl)amino)butyl)-1-methyl-imidazolbis((trifluoromethyl) sulfonyl)amide (TSIL 3)：**

**
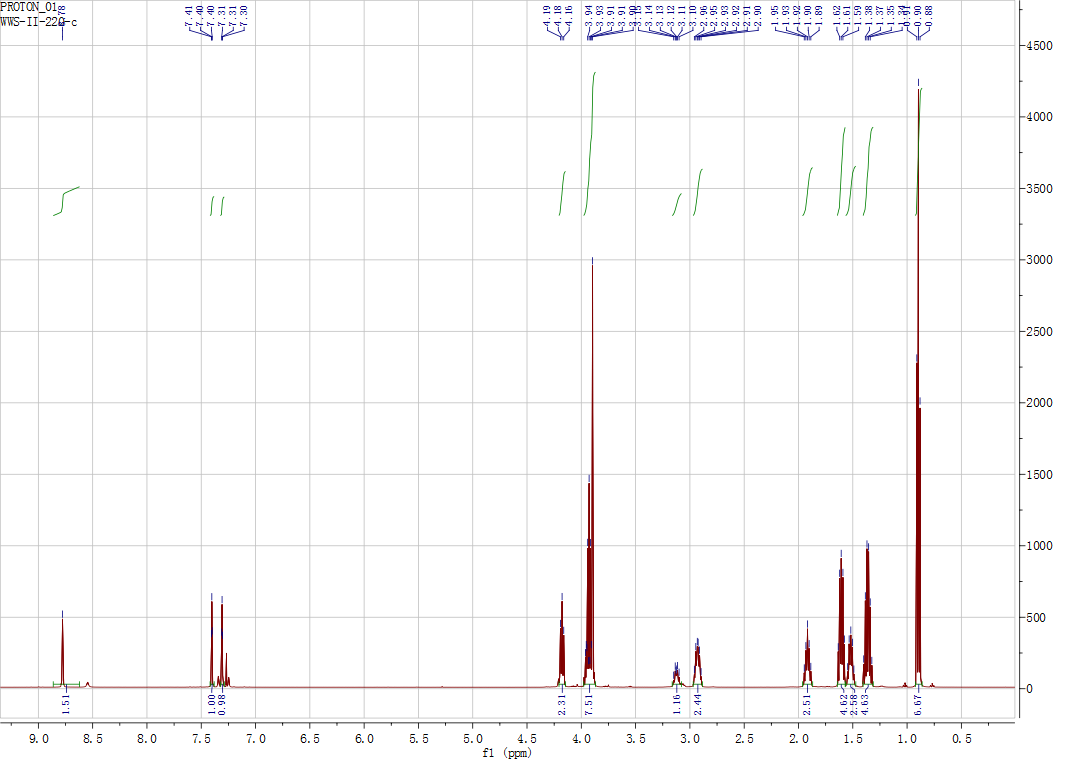
**

**Figure S12.** ^1^H NMR spectrum of TSIL 3.

**
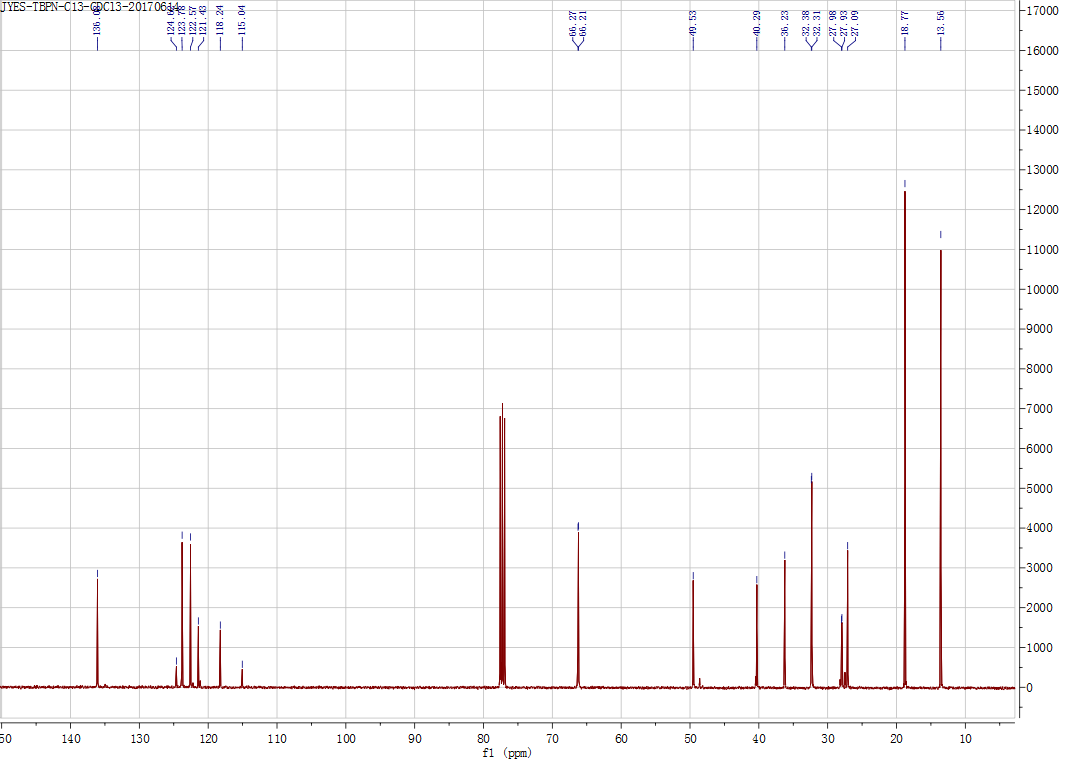
**

**Figure S13.** ^13^C NMR spectrum of TSIL 3.

**
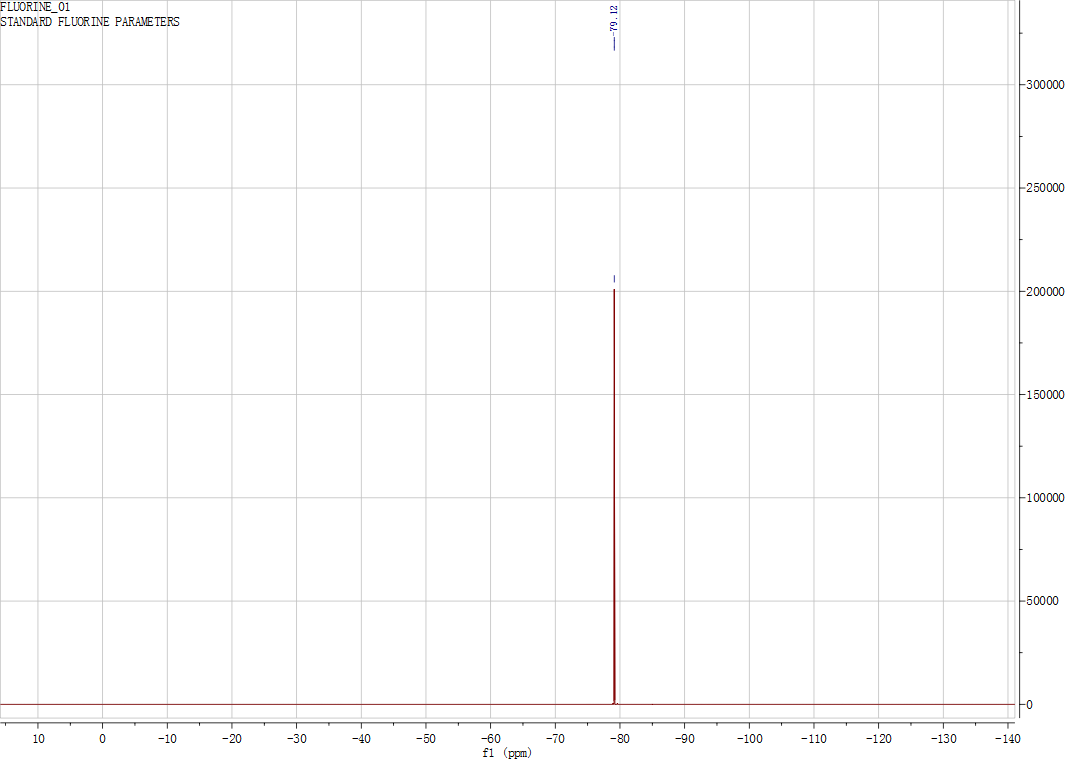
**

**Figure S14.** ^19^F NMR spectrum of TSIL 3.

**
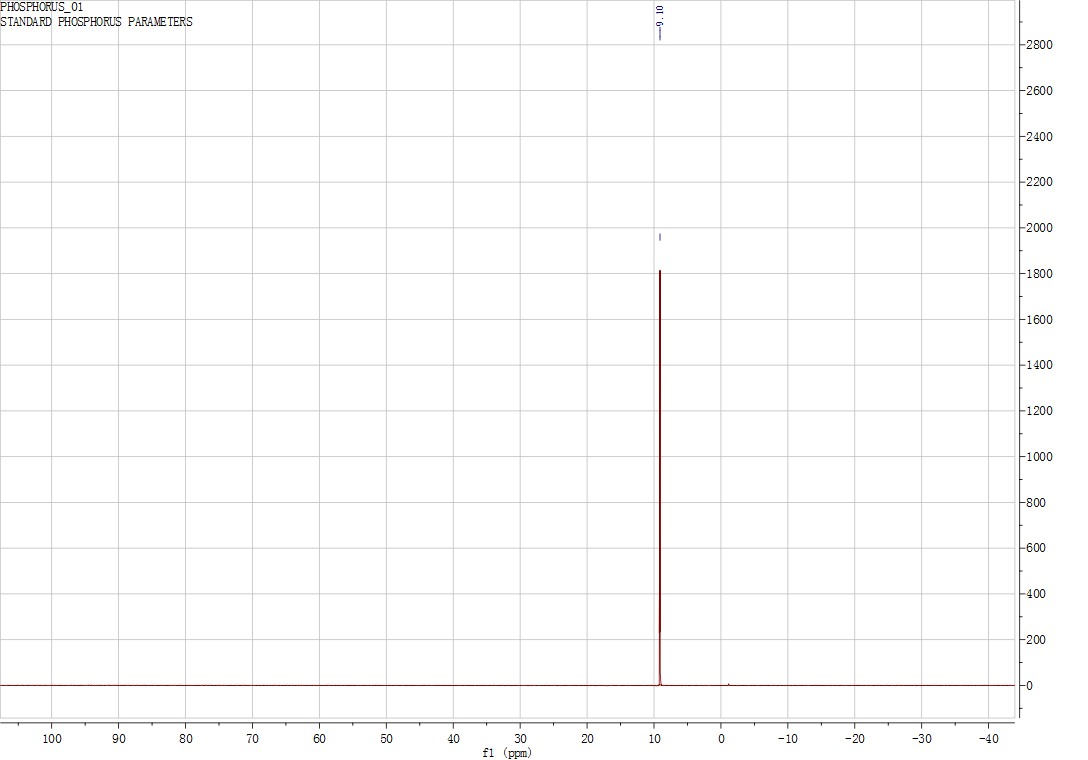
**

**Figure S15.** ^31^P NMR spectrum of TSIL 3.
